# Identifying Functional Genes Influencing *Gossypium hirsutum* Fiber Quality

**DOI:** 10.3389/fpls.2018.01968

**Published:** 2019-01-09

**Authors:** Chengguang Dong, Juan Wang, Yu Yu, Longzhen Ju, Xiaofeng Zhou, Xiaomei Ma, Gaofu Mei, Zegang Han, Zhanfeng Si, Baocheng Li, Hong Chen, Tianzhen Zhang

**Affiliations:** ^1^Key Laboratory of China Northwestern Inland Region, Ministry of Agriculture, Cotton Research Institute, Xinjiang Academy of Agricultural and Reclamation Science, Shihezi, China; ^2^State Key Laboratory of Crop Genetics and Germplasm Enhancement, Nanjing Agricultural University, Nanjing, China; ^3^Department of Agronomy, College of Agriculture and Biotechnology, Zhejiang University, Hangzhou, China

**Keywords:** upland cotton, fiber quality, genome-wide association study, SNP genotyping array, candidate genes

## Abstract

Fiber quality is an important economic index and a major breeding goal in cotton, but direct phenotypic selection is often hindered due to environmental influences and linkage with yield traits. A genome-wide association study (GWAS) is a powerful tool to identify genes associated with phenotypic traits. In this study, we identified fiber quality genes in upland cotton (*Gossypium hirsutum* L.) using GWAS based on a high-density CottonSNP80K array and multiple environment tests. A total of 30 and 23 significant single nucleotide polymorphisms (SNPs) associated with five fiber quality traits were identified across the 408 cotton accessions in six environments and the best linear unbiased predictions, respectively. Among these SNPs, seven loci were the same, and 128 candidate genes were predicted in a 1-Mb region (±500 kb of the peak SNP). Furthermore, two major genome regions (GR1 and GR2) associated with multiple fiber qualities in multiple environments on chromosomes A07 and A13 were identified, and within them, 22 candidate genes were annotated. Of these, 11 genes were expressed [log_2_(1 + FPKM)>1] in the fiber development stages (5, 10, 20, and 25 dpa) using RNA-Seq. This study provides fundamental insight relevant to identification of genes associated with fiber quality and will accelerate future efforts toward improving fiber quality of upland cotton.

## Introduction

Cotton (*Gossypium* spp.) is the most important natural textile fiber source globally. The genus *Gossypium* includes approximately 50 species, 45 diploid (2n = 2x = 26) and 5 tetraploid (2n = 4x = 52). The cultivated types of cotton include two diploids, *G. arboreum* L. and *G. herbaceum* L., and two tetraploids, *G. hirsutum* L. and *G. barbadense* L. Upland cotton (*G. hirsutum* L.) is the most widely cultivated tetraploid cotton species and accounts for 95% of the world’s cotton production because of its high yield and preferable fiber quality, the chromosomes are often numbered in two sets of 13, A1 through A13 and D1 through D13, the genome sizes is approximately 2,500 Mb in the upland cotton (Wendel and Cronn, 2003; Zhang T. et al., 2015). With changes in spinning technology and diverse uses, the improvement of cotton fiber quality is becoming extremely important (Kohel et al., 2001). Especially in recent years, reform of the supply side structure of cotton has been implemented in China and so fiber quality improvement has become the primary objective of cotton breeding programs (Rong et al., 2007). The identification and characterization of genes associated with fiber quality traits is indispensable for both understanding the genetic basis of phenotypic variation and efficient quality trait improvement.

The fiber quality traits of cotton are complex quantitative traits controlled by quantitative trait loci (QTLs). More than 1000 QTLs associated with fiber quality have been detected by the traditional method based on genetic mapping (Said et al., 2013). Association mapping (AM) based on linkage disequilibrium (LD) is a powerful tool to identify genes associated with agronomic traits, and is convenient because it helps avoid the need to screen large biparental mapping populations (Abdurakhmonov, 2007), and can be detect many natural allelic variations simultaneously in a single study (Myles et al., 2009; Lipka et al., 2015). There has been wide use of AM in QTL mapping for important agricultural traits of cotton (Abdurakhmonov et al., 2009; Mei et al., 2013; Cai et al., 2014; Saeed et al., 2014; Zhao et al., 2014; Li et al., 2016; Nie et al., 2016). However, the markers used in AM did not cover the whole cotton genome, due to low marker density. Recent advances in high-throughput sequencing technologies have enabled rapid and accurate resequencing of a large number of genomes (Chia et al., 2012; Huang et al., 2013; Mace et al., 2013; Aflitos et al., 2014). Recently, genome-wide association study (GWAS) based on high-throughput markers have become a higher-resolution and more cost-effective tool for detecting important QTLs or genes associated with complex traits, and have greatly improved resolution and accuracy of genetic mapping (Wang et al., 2011). Application of GWAS to numerous crop species enabled the detection of many QTLs associated with agronomic traits through a large number of single nucleotide polymorphisms (SNPs) (Li et al., 2013, 2017; Lin et al., 2014; Wen et al., 2014; Zhang J. et al., 2015). In cotton, the upland cotton TM-1 genome sequence was released (Li et al., 2015; Zhang T. et al., 2015); and genomic assessment based on the specific-locus amplified fragment sequencing and genome-wide resequencing have provided opportunities to identify required marker coverage in upland cotton (Su et al., 2016a,b; Fang L. et al., 2017; Ma et al., 2018; Su et al., 2018). The specific-locus amplified fragment sequencing and the resequencing can reveal unknown sequence information, but experimental operation and data analysis are complex; comparatively, array-based SNP detection can be used to genotype many samples within a short period and data analysis is relatively easy (Chen et al., 2014). Two commercial high-density cotton SNP arrays were recently designed and used for GWAS analysis (Hulse-Kemp et al., 2015; Cai et al., 2017; Sun et al., 2017; Li et al., 2018; Liu et al., 2018). To better understand the genetic variation of fiber quality at a natural population level, a diverse panel consisting of 408 upland cotton accessions was genotyped by high-density CottonSNP80K array, which was the latest array developed by Nanjing Agricultural University (Cai et al., 2017). The fiber quality traits were measured in six different environments. A GWAS was performed to identify SNP loci or QTL regions associated with fiber quality traits. The candidate genes controlling fiber quality were further predicted by RNA-Seq analysis. These results will lay the foundation for uncovering the genetic variations of complex traits and for fiber quality improvement through breeding by molecular design and genomic selection.

## Materials and Methods

### Plant Accessions and Field Experiments

In this study, a total of 408 upland cotton accessions were selected, comprising 201 accessions that originated from the Northwestern Inland Region (NIR), 88 that originated from the Yellow River region (YRR), 63 from the Yangtze River Region (YtRR), 29 from the northern specific early maturation region (NSEMR) and 27 introduced from abroad (Supplementary Table S1). All 408 accessions were planted at Shihezi (SHZ) in North Xinjiang (85.94°E, 44.27°N) and at Korla (KRL) in South Xinjiang (86.06°E, 41.68°N) for 2013, 2014, and 2015, denoted SHZ13, KRL13, SHZ14, KRL14, SHZ15, and KRL15, respectively. The field experiments were designed so that each accession was grown in a plot with 40–45 plants in two rows, with 0.10 m between plants in rows and 0.45 m between rows. The field experiments used a randomized complete block design with three replications in each environment. The field management conformed to local practices.

### Phenotype Data Collection and Statistical Analyses

The fiber quality traits were measured by the HFT9000 Automatic Fiber Determination System (Premier Inc, Coimbatore, India) at the Key Laboratory of China Northwestern Inland Region, Ministry of Agriculture, China. Fiber quality traits were the upper half mean fiber length (FL, mm), fiber strength (FS, cN/tex), fiber micronaire (FM), fiber uniformity (FU, %) and fiber elongation (FE, %). Software SPSS 19.0 was used to calculate basic statistics and Pearson correlations between traits. Analysis of variance (ANOVA) and broad-sense heritability (*h2 B*) were calculated using Statistical Analysis System (SAS8.0, SAS Institute 1999). The best linear unbiased prediction (BLUP) values for the five fiber quality across the six environments were estimated using the R program, and were used for subsequent GWAS.

### Genotyping and SNP Marker Screening

Total DNA was extracted from young leaves of each accession as described by Paterson et al. (1993). A genome-wide CottonSNP80K genotyping array containing 77,774 SNPs (Cai et al., 2017) was applied to genotype the 408 accessions using the Illumina Infinium platform according to the manufacturer’s protocol (Illumina Inc., San Diego, CA, United States). All SNP genotype data were treated with raw data normalization, clustering and genotype calling by Illumina Genome Studio Genotyping Module (Illumina). The SNPs with a minor allele frequency (MAF) < 0.05 and missing rates ≥ 0.30 were removed to avoid problems of spurious LD and false positive associations. Finally, 48,072 high-quality SNPs were used for GWAS analysis (Supplementary Table S2).

### Population Structure and LD Estimation

The STRUCTURE version 2.2 (Pritchard et al., 2000) was used to assess the population structure among 408 accessions, based on Bayesian MCMC set to 10,000, burn-in set to a running time of 100,000 and *K*-value set to 1–9, with the rest of the parameters using software default settings. The most likely number of clusters (*K*) was selected by comparing the logarithmic probabilities of Ln*P*(D) and Δ*K* data as previously described by Evanno et al. (2005). Nei’s genetic distances among the 408 accessions were calculated with Phylip software (version 3.69) (Felsenstein, 1989), and a phylogenetic tree was constructed with Dendroscope software (Huson et al., 2007). Principal component analysis (PCA) using EIGENSTRAT software (Price et al., 2006) was used to further verify population structure. The LD parameter (*r*^2^) between linked SNPs was estimated using Haploview software (Barrett et al., 2005). The core parameters were set as follows: mingeno 0.6, minmaf 0.05, and hwcutoff 0.001. The LD decay rate was measured as the chromosomal distance at which the average pairwise correlation coefficient dropped to half of its maximum value (Huang et al., 2010). The LD decay map was drawn using the R program.

### GWAS and Elite Allele Identification

The BLUPs for the six environments and the phenotypic values of five fiber quality traits for each environment were used in GWAS with the Efficient Mixed-Model Association Expedited program (EMMAx), the threshold adopted was the Bonferroni *P* ≤ 1/n [1/48,072 = 2.08 × 10^-5^, -log_10_(*P*) ≥ 4.68] (Cai et al., 2017; Sun et al., 2017) to determine significant associations, where n is the number of SNP markers used in the association analysis. Manhattan plots were performed using the R software package “qqman”. For the significant marker–trait associations, elite haplotypes were identified according to the breeding objectives of the target trait. The formula for calculating phenotypic effect (*a_i_*) of alleles was described by Zhang et al. (2013). For FL, FS, FU and FE, *a_i_* > 0 indicated an elite allele; and for FM, *a_i_* < 0 indicated an elite allele. Box plots for phenotypic differences of traits among different haplotypes were constructed using SPSS19.0.

### Prediction of Candidate Genes in GWAS-Associated Loci

According to the GWAS-associated SNP markers, candidate genes were identified within 500 kb upstream and downstream of peak SNPs. Gene functions were predicted based on the expression profiling data in different TM-1 tissues (the SRA database: PRJNA248163; Zhang T. et al., 2015): root, stem, leaf, calycle, fiber-5 DPA (fiber at 5 days post-anthesis), fiber-10 DPA, fiber-20 DPA and fiber-25 DPA^1^. Normalized fragments per kilobase of transcript per million fragments mapped (FPKM) values were calculated to predict the expression levels of these candidate genes. The calculation method for FPKM was as follows: FPKM = cDNA fragments/Mapped Reads (Millions) × Transcript Length (Kb). Further gene annotations were performed from by gene ontology (GO) items on the cotton website^2^.

## Results

### Phenotypic Variations in Six Environments

Fiber quality performance in six environments and the BLUPs in the 408 accessions are presented in Supplementary Table S3 and Supplementary Figure S1. The FL, FS, FM, FU, and FE were in the ranges of 21.20–36.70 mm, 23.70–43.60 cN/tex, 2.60–6.00, 78.90–88.40, and 5.90–7.60%, respectively. The ANOVA showed that the effects of genotype (G) and genotype × environment (G × E) interactions were significant (*P* < 0.01) on the five traits except for FU and FE (*P* < 0.05). The *h2 B* values for the five traits were in the range of 69.54–91.05%, with the maximum *h2 B* for FL. The correlations between the five fiber quality traits using the BLUP results showed that FL was significantly positively correlated with FS, FU, and FE, and a positive correlation was also found for FS with FU and FE. There were significant negative correlations of FM with FL and FS (Supplementary Table S4). These results demonstrated that cotton fiber quality traits were highly related to each other, and provide meaningful theoretical knowledge for cotton fiber quality breeding.

### Genetic Variation and Population Structure

**FIGURE 1:**
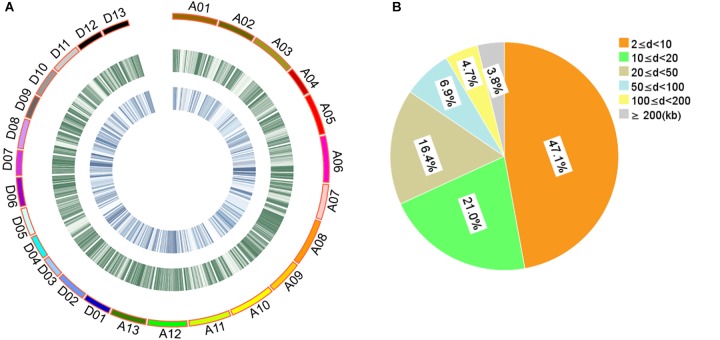
Description of SNPs and genetic diversity of 408 cotton accessions. **(A)** Genetic diversity of 408 cotton genomes. The serial numbers of 26 chromosomes are represented by different colors. From the outer to the inner circle, the curves depict SNP density (the number of SNPs per 100-kb window) and the level of genetic differentiation (the *F_ST_* values per 100-kb window) between G1 and G2, respectively. **(B)** Proportion of the 48,072 SNPs categorized by the adjacent SNP distances. “d” represents the distance between two adjacent SNPs.

The 408 cotton accessions were genotyped using the CottonSNP80K Illumina Infinium SNP array at Beijing Compass Biotechnology Co. Ltd. (Beijing, China). After removing MAF < 0.05 and missing rates ≥ 0.30, a total of 48,072 SNPs of high genotyping quality (48,072/77,774, 61.81%) were used for subsequent GWAS (Supplementary Table S5 and Figure 1A). The 48,072 SNPs covered all 26 chromosomes, with 25,444 and 22,628 SNPs in the A and D subgenomes, respectively. The highest number of SNPs was identified on chromosome A08 (3408 SNPs) and the least on chromosome D04 (910 SNPs). The average SNP density was approximately one SNP per 42.88 kb, with the highest density on chromosome D07 and the least on chromosome A02 (one SNP per 69.31 and 22.89 kb, respectively). For these SNPs, 84.55% of the adjacent SNP distances were < 50 kb and only 0.92% were > 500 kb (Figure 1B).

**FIGURE 2:**
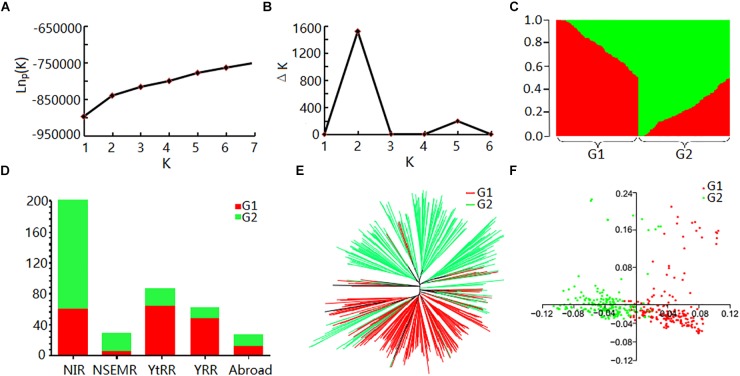
Analysis of population structure of 408 upland cotton accessions. **(A)** Mean Ln*P*(D) values plotted as the number of subgroups; **(B)** Δ*K* values plotted as the number of subgroups; **(C)** population structure based on STRUCTURE when *K* = 2; **(D)** distribution of accessions in different cotton regions of two subgroups. Red and green represent G1 and G2, respectively; **(E)** NJ tree based on Nei’s genetic distances; **(F)** principal component analysis.

Evaluation of the population structure of the 408 cotton accessions by STRUCTURE showed that the Ln*P*(D) value corresponding to each putative *K* kept increasing with *K-*value and did not show an inflection point (Figure 2A). The Δ*K* value showed a maximum likelihood at *K* = 2 (Figure 2B), suggesting that the 408 cotton accessions could be divided into two major subgroups (Supplementary Table S6 and Figure 2C): G1 and G2. Each subgroup was composed of accessions from different ecological zones, and was unrelated to geographical distribution (Figure 2D). Furthermore, phylogenetic analysis (Figure 2E) and PCA (Figure 2F) also agreed well with the clustering results in STRUCTURE. Moreover, the genetic diversity across the 26 chromosomes (Figure 1A and Supplementary Table S7), shown by *F_ST_* values of the two subgroups of 0.094, indicated a low level of subpopulation differentiation or a low level of genetic diversity during cotton domestication. The decay of LD with physical distance between SNPs was approximately 0.50 Mb, with *r^2^* = 0.03 at half of the maximum value (Supplementary Figure S2).

### Genome-Wide Association Study

**FIGURE 3:**
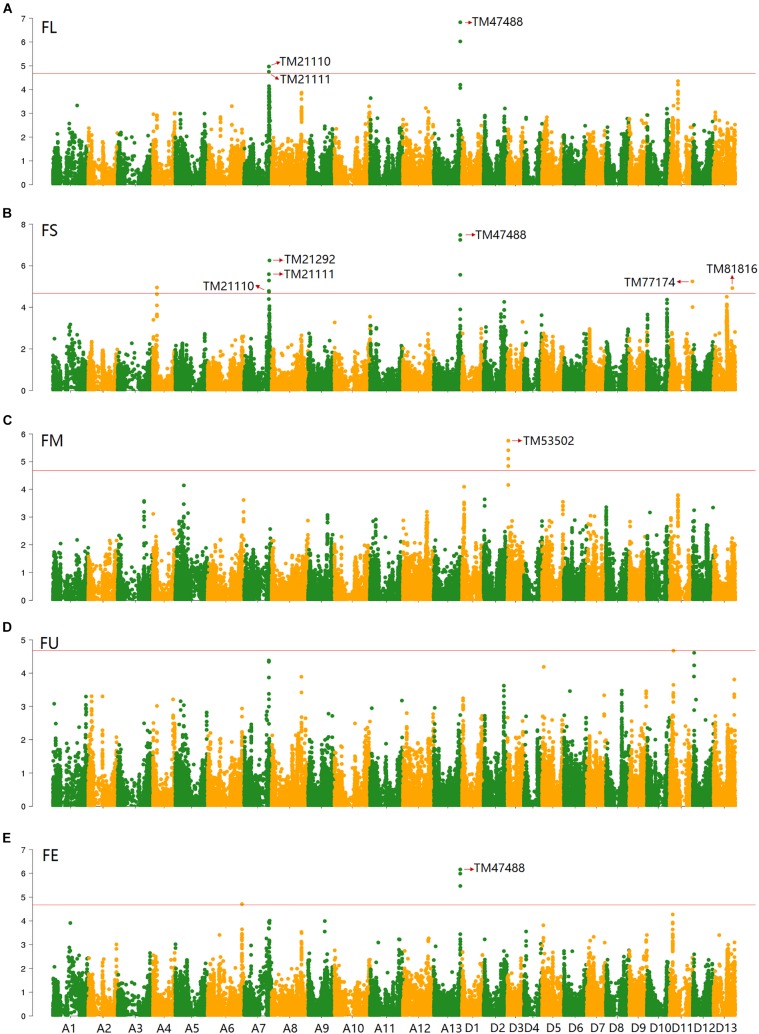
GWAS of the FL **(A)**, FS **(B)**, FM **(C)**, FU **(D)**, and FE **(E)** in the BLUPs using EMMAX. The horizontal line indicates the threshold (4.68). Seven SNPs (the same loci between 30 significant SNPs and 23 SNPs across the BLUPs) are indicated by the red arrow in the Manhattan plots.

Based on the genotypic data of 48,072 high-quality SNPs, phenotypic mean values of different environments and the BLUPs, the GWAS was performed to identify the associated loci in 408 cotton accessions. A total of 90 significant SNPs were identified for the five fiber quality traits in SHZ and KRL for 2013–2015 (Supplementary Table S8). The numbers of loci associated with FL, FS, FM, FU, and FE were 29, 17, 8, 22, and 14, respectively. To select major loci among all significant SNPs, the SNPs with the lowest *P*-values (the highest log_10_*P*) were maintained within a 1.0-Mb window according to the average LD decay rate in the panel. Finally, 30 significant SNPs were identified for these five traits in different environments (Supplementary Table S8). For FL, six peak SNPs were distributed on chromosomes A05, A07, A13, and D11, and 93 candidate genes were predicted within 1.0 Mb (±500 kb of the peak SNP) (Supplementary Table S8 and Supplementary Figure S3). For FS, seven peak SNPs were distributed on chromosomes A04, A07, A13, D11 and D13, and 129 candidate genes predicted within 1.0 Mb (Supplementary Table S8 and Supplementary Figure S4). For FM, three peak SNPs were distributed on chromosomes A12, D03 and D11, and 38 candidate genes predicted within 1.0 Mb (Supplementary Table S8 and Supplementary Figure S5). For FU, six peak SNPs were distributed on chromosomes A07, A08, A09, D03, and D13, and 108 candidate genes predicted within 1.0 Mb (Supplementary Table S8 and Supplementary Figure S6). For FE, eight peak SNPs were distributed on chromosomes A01, A07, A09, A11, A13, and D09, and 90 candidate genes predicted within 1.0 Mb (Supplementary Table S8 and Supplementary Figure S7). We also found that more candidate genes for fiber quality were predicted in the A (302) than the D subgenome (156). Moreover, 23 significant SNPs were detected across the BLUPs, and 164 candidate genes were predicted in the CottonSNP80K array (Supplementary Table S9). Most importantly, seven SNPs were the same loci for 30 significant SNPs and 23 SNPs across the BLUPs (Figure 3), and 128 candidate genes were predicted within 1.0 Mb (Table 1 and Supplementary Tables S10, S11). Furthermore, we observed that the expression of 91 genes of them were expressed [log_2_(1 + FPKM) > 1] in the fiber development stages (5, 10, 20, and 25 dpa) using RNA-Seq. Gene ontology (GO) analysis indicated that the functions of most genes were binding and enzymatic activity belong to molecular functions category. This information provides a convenient way to rapidly identify candidate genes associated with fiber quality.

**Table 1:** Summary of seven significant peak SNPs associated with fiber quality traits and candidate genes within 500 kb either side of the SNP locus.

SNPs	Chromosome	Site	Traits	Environment	No. of candidate gene
TM21110	A07	70483507	FL	BLUPs, KRL13, KRL14	8
			FS	BLUPs, KRL13, KRL14	
TM21111	A07	70492663	FL	BLUPs, KRL13, KRL14	1
			FS	BLUPs, KRL13, KRL14	
TM47488	A13	75014691	FL	BLUPs, SHZ13, SHZ14, KRL13, KRL14	43
			FS	BLUPs, SHZ13, SHZ14, KRL13, KRL14	
			FE	BLUPs, SHZ13	
TM21292	A07	72067994	FS	BLUPs, SHZ13, SHZ14, SHZ15, KRL13, KRL14	16
TM77174	D11	64945961	FS	BLUPs, SHZ14, KRL13	19
TM81816	D13	52852792	FS	BLUPs, SHZ14	18
TM53502	D03	2481487	FM	BLUPs, SHZ13	23


**FIGURE 4:**
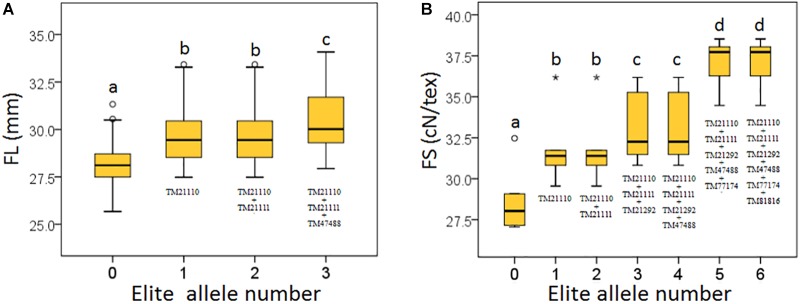
Build-up effect analysis for different numbers of elite alleles. **(A)** FL, fiber length; **(B)** FS, fiber strength. The *X*-axis represents the number of elite alleles carried by the accessions and the *Y*-axis represents trait mean value. Different lowercase letters above the plots represent Duncan’s multiple comparison at *P* < 0.05.

Based on the seven significant peak SNPs, each SNP locus could be classified into three alleles; according to cotton quality breeding objectives, for FL, FS, FU, and FE, the alleles with positive effects are elite alleles. For FM, the alleles with negative effects were elite alleles. The effects of elite alleles for the five traits directly related to fiber quality are summarized in Table 2. These findings indicated that the phenotypic characteristics with elite alleles displayed a significantly average values than the other non-included elite alleles (Supplementary Figure S8), and further confirmed that there were major QTLs controlling fiber quality in the regions adjacent to the seven associated SNP loci. In addition, three SNP loci (TM21110, TM21111, and TM47488) were associated with FL, and six SNP loci (TM21110, TM21111, TM21292, TM47488, TM77174, and TM81816) were associated with FS, without taking into account the effect of interactions among these loci, with the accumulation of more elite alleles in accessions, with the larger average phenotypic value significant increased (Figure 4). These results indicated that the genetic control of fiber quality generally had additive effects in cotton. Consequently, increasing the frequency of elite alleles would significantly improve cotton fiber quality.

**Table 2:** Summary of elite alleles and phenotypic effects.

Trait	SNP	Chromosome	Genotype	Elite allele	ANOVA *P* (elite-unelite)	*a_i_*
FL	TM21110	A07	A/G	GG	5.03E-13^∗∗∗^	1.44
	TM21111	A07	T/C	CC	2.51E-12^∗∗∗^	1.35
	TM47488	A13	T/C	CC	1.37E-12^∗∗∗^	0.96
FS	TM21110	A07	A/G	GG	2.93E-15^∗∗∗^	2.65
	TM21111	A07	T/C	CC	3.18E-15^∗∗∗^	2.53
	TM21292	A07	T/C	CC	3.07E-13^∗∗∗^	3.01
	TM47488	A13	T/C	CC	9.75E-18^∗∗∗^	1.91
	TM77174	D11	A/G	GG	0.0031^∗∗^	3.73
	TM81816	D13	A/G	GG	1.54E-15^∗∗∗^	0.85
FM	TM53502	D03	A/C	AA	4.05E-09^∗∗∗^	-0.05
FE	TM47488	A13	T/C	CC	2.06E-11^∗∗∗^	0.06


*a_i_, positive phenotypic effect value. The formula for calculating: a_i_ = Σx_ij_/n_i_ – ΣN_k_/n_k_*

*where a_i_ represents the phenotypic effect of the ith allele, x_ij_ represents the jth material’s phenotypic value of the ith allele, n_i_ represents the number of materials having the ith allele; N_k_ represents the nth material’s phenotypic value of all materials, and n_k_ is the number of materials. ^∗∗^, ^∗∗∗^ Significant differences at P < 0.01 and P < 0.001, respectively*

### Identification of Two Genome Regions Pleiotropically Improving Fiber Qualities

**FIGURE 5:**
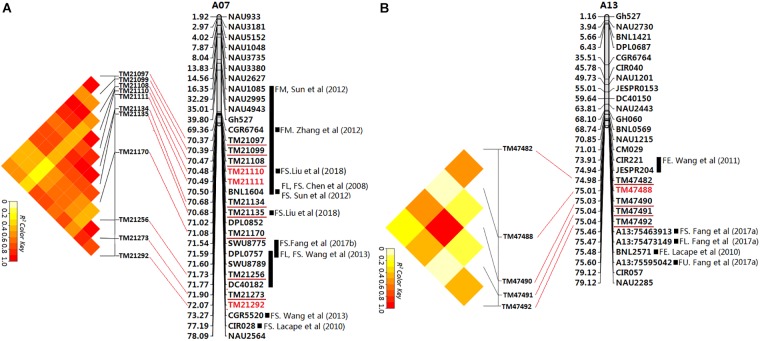
Physical map of SSR markers identified by electronic PCR based on the reference genome sequence (Zhang T. et al., 2015). The SSR markers linked to fiber quality QTLs from previous studies. The unit of physical distance for the chromosomes is Mb; gray columnar graph represents SSR markers or QTLs from previous studies in the region adjacent to the associated SNP loci identified in this study. **(A)** The first major genome region (GR1) on chromosome A07; **(B)** The second major genome region (GR2) on chromosome A13.

Gene linkage and pleiotropy are common phenomena in crop breeding. In this study, out of the seven significant peak SNPs, three showed signal linkage or pleiotropy associated with multiple traits in the six environments. In particular, two major genome regions including 11 and five loci (GR1 and GR2) on chromosomes A07 and A13 were associated with multiple fiber qualities in multiple environments (Figure 5). The TM21110 and TM21111 loci on chromosome A07 were associated with both FL and FS in KRL13, KRL14 and the BLUPs. Moreover, the correlation between FL and FS (*r* = 0.848, *P* < 0.01) was significant in the BLUPs (Supplementary Table S4). The TM47488 locus on chromosome A13 was closely related to FL, FS and FE in SHZ13, SHZ14, KRL13, KRL14 and the BLUPs. These associated loci may explain the correlations among fiber qualities: between FL and FS (*r* = 0.848, *P* < 0.01), FL and FE (*r* = 0.829, *P* < 0.01) and FS and FE (*r* = 0.748, *P* < 0.01) in the BLUPs (Supplementary Table S4).

The first major genome region of 1.70 Mb (GR1, within 70,365,245–72,067,994 bp) on chromosome A07 including 11 loci (TM21097, TM21099, TM21108, TM21110, TM21111, TM21134, TM21135, TM21170, TM21256, TM21273, and TM21292) was associated with FL, FS, and FE in different environments and the BLUPs (Supplementary Table S8), An LD block analysis of the 11 loci showed that these markers had high levels of LD (Figure 5A). Using the CottonSNP80K array information, 16 candidate genes were annotated within the GR1, and their functional annotations are summarized in Table 3. RNA-Seq analysis from eight upland cotton TM-1 tissues (Zhang T. et al., 2015) of the 16 genes showed that six (*Gh_A07G1723, Gh_A07G1727, Gh_A07G1731, Gh_A07G1742, Gh_A07G1751*, and *Gh_A07G1752*) were expressed [log_2_ (1 + FPKM) > 1] in the fiber. Notably, *Gh_A07G1723* and *Gh_A07G1752* was preferentially expressed in fiber of 5 DPA, and *Gh_A07G1731* was mainly expressed in fiber of 10 DPA (Table 3 and Supplementary Table S11). The second major genome region of 65.09 kb (GR2, within 74,977,977–75,043,070 bp) on chromosome A13, including five loci (TM47482, TM47488, TM47490, TM47491, and TM47492), were associated with FL, FS and FE in different environments and the BLUPs (Supplementary Table S8), An LD block analysis of the five loci showed that these markers had high levels of LD (Figure 5B). Use of the CottonSNP80K array information allowed six genes to be annotated within the GR2 region, and their functional annotations are summarized in Table 3. RNA-Seq analysis showed that of the five genes (*Gh_A13G1614, Gh_A13G1615, Gh_A13G1616, Gh_A13G1619*, and *Gh_A13G1622*) expressed [log_2_(1 + FPKM) > 1] in the fiber, which *Gh_A13G1615* was mainly expressed in fiber of 20 DPA (Table 3 and Supplementary Table S11). Generally, these results suggested that the above candidate genes played important roles in different stages of fiber development.

**Table 3:** The 22 candidate genes with fiber quality traits.

Chromosome	Gene name	Gene_start	Gene_end	Annotation	Expression level
A7	*Gh_A07G1723*	70380260	70385380	Heat shock protein 90-like	>1
A7	*Gh_A07G1724*	70401196	70401555	Unknown	
A7	*Gh_A07G1727*	70458716	70466147	Nucleoporin, Nup133/Nup155-like	>1
A7	*Gh_A07G1730*	70665067	70669318	Transducin/WD40 repeat-like superfamily protein	
A7	*Gh_A07G1731*	70711596	70712168	Unknown	>1
A7	*Gh_A07G1735*	70778523	70779569	Snf1-related protein kinase regulatory subunit beta-2	
A7	*Gh_A07G1742*	70985378	70986640	FASCICLIN-like arabinogalactan 2	>1
A7	*Gh_A07G1744*	71156295	71158111	Cytochrome p450 79a2	
A7	*Gh_A07G1745*	71430100	71431232	emp24/gp25L/p24 family/GOLD family protein	
A7	*Gh_A07G1750*	71570464	71571897	Receptor like protein 45	
A7	*Gh_A07G1751*	71574475	71580347	Nudix hydrolase homolog 23	>1
A7	*Gh_A07G1752*	71640549	71640998	RING/U-box superfamily protein	>1
A7	*Gh_A07G1755*	71682050	71682979	Plant protein of unknown function (DUF868)	
A7	*Gh_A07G1756*	71683734	71690864	Unknown	
A7	*Gh_A07G1757*	71951926	71957589	Subtilisin-like serine endopeptidase family protein	
A7	*Gh_A07G1761*	72009668	72010690	Unknown	
A13	*Gh_A13G1614*	74974260	74978836	Cyclic nucleotide gated channel 5	>1
A13	*Gh_A13G1615*	74979629	74982299	Endomembrane protein 70 protein family	>1
A13	*Gh_A13G1616*	74984317	74990193	Cytosol aminopeptidase family protein	>1
A13	*Gh_A13G1619*	75012308	75016124	Fatty acid desaturase 6	>1
A13	*Gh_A13G1621*	75029006	75040535	Actin binding	
A13	*Gh_A13G1622*	75042666	75046219	Reticulon family protein	>1


*>1 indicates expression of the genes that log_2_(FPKM + 1) > 1 in fiber different development stages among eight upland cotton (TM-1) tissues.*

## Discussion

Natural fiber products are favored by the majority of consumers because of moisture absorption, air permeability and warmth retention. Cotton fiber is the most important raw material in the textile industry. Its quality is directly related to the quality and grade of the textile products, and improving fiber quality has always been the primary objective of cotton breeding programs. In this study, a GWAS was performed based on a large natural population of upland cotton and the high-density CottonSNP80K array. First of all, five of the most important fiber quality traits of breeding were accurately measured in six different environments. Phenotypic trait analysis based on the BLUP results preferably eliminated environmental effects and improved the accuracy of predicting complex quantitative traits. This work provided accurate phenotypic data for association analysis. Furthermore, we conducted population structure analysis and LD estimation based on the high-density CottonSNP80K array. The panel was classified into two groups based on three different analysis methods (Figure 2). The clustering results showed no obvious correspondence with geographical distribution, similar to previous results (Wang et al., 2016; Sun et al., 2017). The *F*_ST_ values were calculated between G1 and G2, and were lower than in maize (Jiao et al., 2012) and hexaploid wheat (Cavanagh et al., 2013). These results supported the narrow diversity of upland cotton cultivars and that most were derived from the same ancestral strain (Fang L. et al., 2017). We also found that the approximate LD decay was 500 kb in this panel (Supplementary Figure S2), consistent with previous studies in which upland cotton had a long-range LD decay distance from ∼500 to ∼800 kb (Cai et al., 2017; Sun et al., 2017), which provided a good reference for further selecting of candidate genes. Eventually, we found that two major genome regions including 16 SNP loci were associated with multiple fiber qualities in multiple environments, and 22 candidate genes were annotated. This study provided numerous new genomic resources for further understanding the genetic basis of fiber quality and so to improve upland cotton.

Presently, many QTL related to cotton fiber qualities have been mapped, and some fiber quality QTL hotspots have been discovered by a comparative meta-analysis (Said et al., 2015). Similarly, a few associated SNPs with fiber quality have been detected via GWAS in upland cotton (Sun et al., 2017; Li et al., 2018; Liu et al., 2018; Ma et al., 2018). In this study, We compared the GR1 and GR2 on chromosomes A07 and A13 detected in our GWAS (Figure 5) with SSRs and SNPs associated with QTLs for the fiber quality traits identified in previous studies by electronic PCR based on the reference genome sequence (Zhang T. et al., 2015). In the GR1 (Figure 5A), previous researchers found 10 QTLs (Chen et al., 2008; Lacape et al., 2010; Sun et al., 2012; Wang et al., 2013; Zhang et al., 2012; Fang X. et al., 2017) associated with fiber quality within or adjacent regions through linkage analysis and QTL mapping, Liu et al. (2018) detected 10 SNP loci associated with FS in the GR1 region by GWAS, and two SNP loci were the same loci with this study (TM21110 and TM21135), Sun et al. (2017) and Ma et al. (2018) have identified a number of cluster_A07 SNPs for FS are distributed in genome region A07: 71.99–72.25 Mbp, There was partial overlap with GR1 (70.37–72.07 Mbp) identified in this study. These results further indicated that the GR1 is an important hotspot controlling fiber quality. In the GR2 (Figure 5B), previous researchers also found two QTLs (Lacape et al., 2010; Wang et al., 2011) associated with fiber quality in the adjacent regions through linkage analysis, Fang L. et al. (2017) detected three SNP loci associated with fiber quality in the GR2 adjacent region by GWAS, these associated QTLs or SNPs were excluded in the GR2 of the previous reports. Therefore, the GR2 may be novel hotspot controlling fiber quality.

Cotton fiber development is a complex process involving initiation, elongation (primary cell wall synthesis), secondary cell wall synthesis and maturation (Kim and Triplett, 2001). Previous research indicated that many genes regulate different fiber developmental phases. In our study, 22 candidate genes in GR1 and GR2 on chromosomes A07 and A13 were annotated, and 11 genes showed higher expression in different periods of fiber development (Table 3). For example, gene *Gh_A07G1723* in GR1 belonged to the Arabidopsis heat shock protein 90-like, and this protein serves as a molecular chaperone with important roles in plant cellular signal transduction pathway and influences plant growth and development (Ishiguro et al., 2002; Sangster et al., 2007). Interestingly, of four genes (*Gh_A07G1748, Gh_A07G1749, Gh_A07G1750, and Gh_A07G1751*) reported in GR1 of upland cotton, one is engaged in mediating a combination of cell elongation and SCW biosynthesis during cotton fiber development (Fang X. et al., 2017). In addition, two genes (*Gh_A13G1606 and Gh_A13G1624*) in GR2 neighboring regions had higher expression levels for fiber-20 DPA and fiber-5 DPA than in other tissues (Supplementary Figure S9), *Gh_A13G1606* on chromosome A13, a homologous of AT4G32330 locus for protein WAVE-DAMPENED 2-like 6 (WVD2) in Arabidopsis, was identified as an activation-tagged allele that can cause plant organ stockiness, which is critical for the organization of plant microtubules (Perrin et al., 2007). The *Gh_A13G1624*, a homolog of Arabidopsis At4g25140, belongs to the S12E family of ribosomal proteins and plays an important role in intracellular protein biosynthesis (Maguire and Zimmermann, 2001; Khairulina et al., 2010). However, the biological validation of these candidate genes in developing cotton fibers needs further research.

## Conclusion

In this study, based on a high-density CottonSNP80K array, a total of 30 and 23 significant SNPs associated with five fiber quality traits were identified across the 408 cotton accessions in six environments and the BLUP, respectively. Among these SNPs, seven loci were the same, and 128 candidate genes were predicted in a 1-Mb region. Most importantly, two major genome regions (GR1 and GR2) associated with multiple fiber qualities in multiple environments on chromosomes A07 and A13 were identified, and 22 candidate genes were annotated. This study provides fundamental insight relevant to identification of genes associated with fiber quality and will accelerate future efforts toward improving fiber quality of upland cotton.

## Author Contributions

TZ and HC designed and supervised the research. CD, JW, YY, XZ, XM, and BL conducted the field trials to evaluate the traits. CD, JW, LJ, GM, ZH, and ZS performed the data analysis. CD and JW wrote the manuscript. All of the authors read and approved the manuscript.

## Conflict of Interest Statement

The authors declare that the research was conducted in the absence of any commercial or financial relationships that could be construed as a potential conflict of interest.

## Footnotes

1 https://cottonfgd.org/

2 http://www.ebi.ac.uk/

## Supplementary Material

Figure S1 Frequency distributions based on the BLUPs of the five fiber quality traits in six environments. **(A)** fiber length (FL); **(B)** fiber strength (FS); **(C)** fiber micronaire value (FM); **(D)** fiber uniformity (FU); and **(E)** fiber elongation (FE).

Figure S2 Average LD decay estimated in 408 cotton accessions.

Figure S3 Manhattan plots showing the GWAS for FL in six environments. The horizontal line indicates the threshold (–log_10_*P* > 4.68).

Figure S4 Manhattan plots showing the GWAS for FS in six environments. The horizontal line indicates the threshold (–log_10_*P* > 4.68).

Figure S5 Manhattan plots showing the GWAS for FM in six environments. The horizontal line indicates the threshold (–log_10_*P* > 4.68).

Figure S6 Manhattan plots showing the GWAS for FU in six environments. The horizontal line indicates the threshold (–log_10_*P* > 4.68).

Figure S7 Manhattan plots showing the GWAS for FE in six environments. The horizontal line indicates the threshold (–log_10_*P* > 4.68).

Figure S8 Boxplots depicting the genetic effects of SNPs with significant associations with fiber quality traits. **(A)** Boxplot diagram depicting the genetic effect of three significant SNPs to FL. **(B)** Boxplot diagram depicting the genetic effect of six significant SNPs to FS. **(C)** Boxplot diagram depicting the genetic effect of one significant SNP to FM. **(D)** Boxplot diagram depicting the genetic effect of one significant SNP to FE. The box shows the lower quartile, median and upper quartile values, and the whiskers show the range of phenotypic variation in the population.

Figure S9 Expression levels of two genes associated with fiber quality traits. **(A)** Gh_A13G1606, **(B)** Gh_A13G1624.

Table S1 The ecological origins information of the 408 Upland cotton accessions.

Table S2 The Genotyping data of 408 materials from high-density array.

Table S3 Descriptive statistics and ANOVA for five fiber quality traits.

Table S4 Correlation analysis of five fiber quality traits.

Table S5 Genome-wide SNP distributions on the 26 chromosomes of the 408 upland cotton accessions.

Table S6 Subpopulation arrangement of the 403 upland cotton accessions.

Table S7 Levels of genetic differentiation on the 26 chromosomes.

Table S8 The summary of significant SNPs and candidate genes for fiber quality traits in six environments.

Table S9 The summary of significant SNPs and candidate genes for fiber quality traits in the BLUPs.

Table S10 Hundred and twenty eight candidate genes with fiber quality traits.

Table S11 Transcriptome sequencing data of 128 candidate genes from 8 upland cotton (TM-1) tissues.
