# Supplementary figures and images for: Identifying Functional Genes Influencing Gossypium hirsutum Fiber Quality
Source: Front Plant Sci. 2019 Jan 9;9:1968. doi: 10.3389/fpls.2018.01968 (PMC6334163; doi:10.3389/fpls.2018.01968)

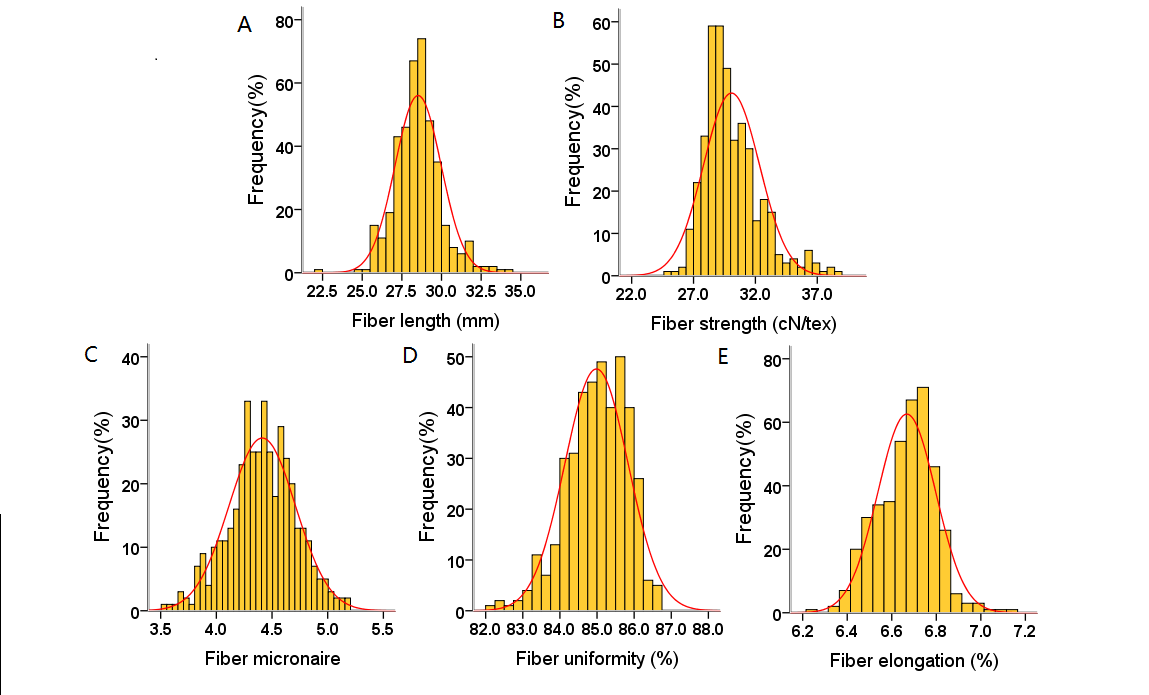

Supplement: Figure S1 — Frequency distributions based on the BLUPs of the five fiber quality traits in six environments. (A) fiber length (FL); (B) fiber strength (FS); (C) fiber micronaire value (FM); (D) fiber uniformity (FU); and (E) fiber elongation (FE). [file Image_1.TIF]

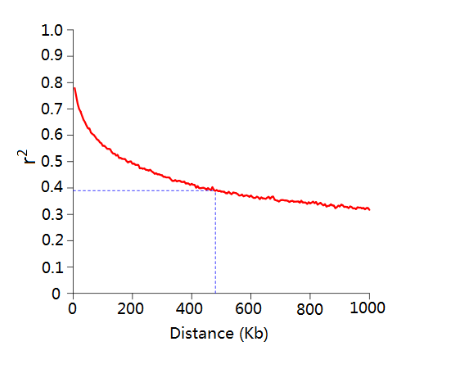

Supplement: Figure S2 — Average LD decay estimated in 408 cotton accessions. [file Image_2.TIF]

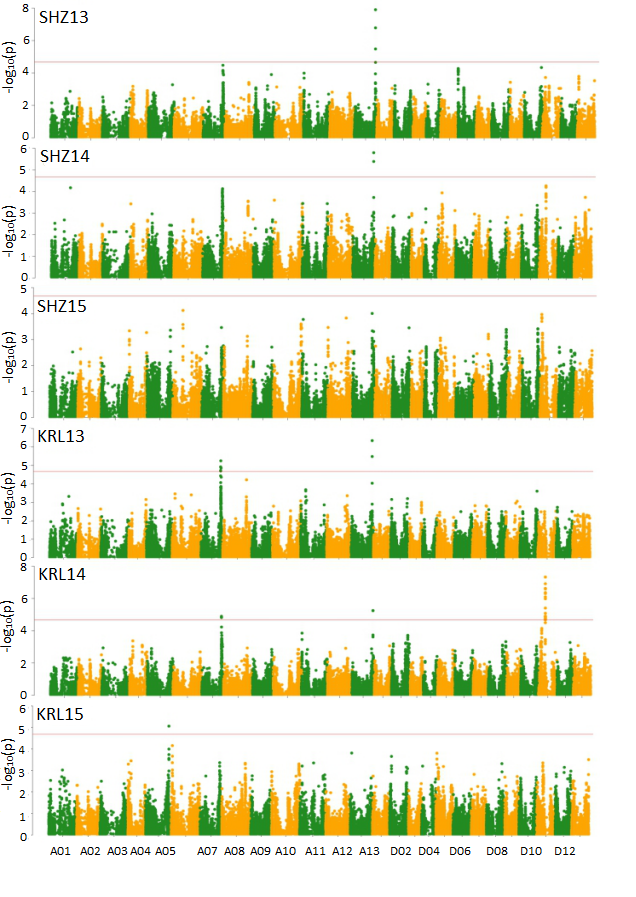

Supplement: Figure S3 — Manhattan plots showing the GWAS for FL in six environments. The horizontal line indicates the threshold (–log10P > 4.68). [file Image_3.TIF]

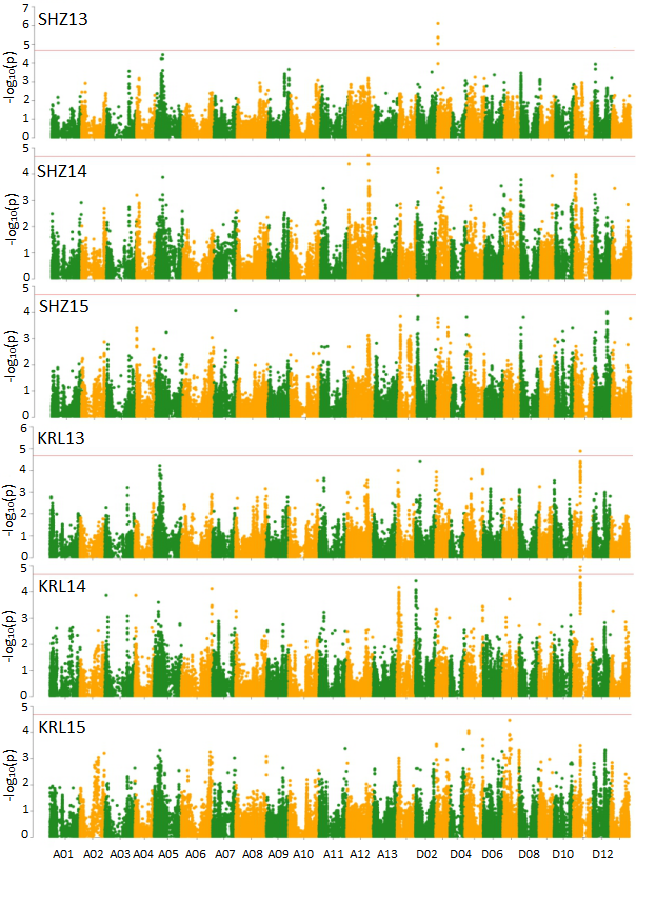

Supplement: Figure S4 — Manhattan plots showing the GWAS for FS in six environments. The horizontal line indicates the threshold (–log10P > 4.68). [file Image_4.TIF]

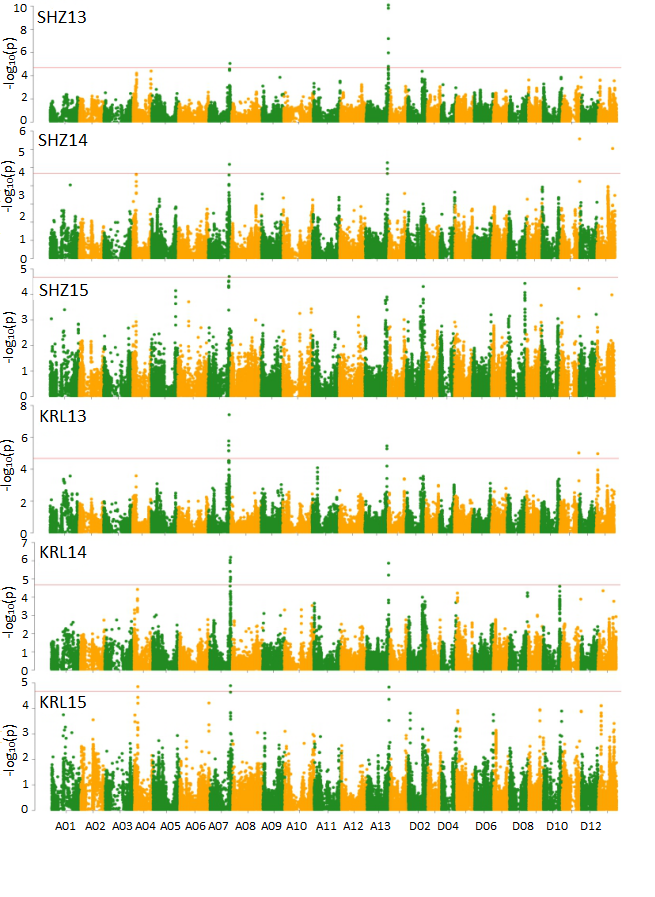

Supplement: Figure S5 — Manhattan plots showing the GWAS for FM in six environments. The horizontal line indicates the threshold (–log10P > 4.68). [file Image_5.TIF]

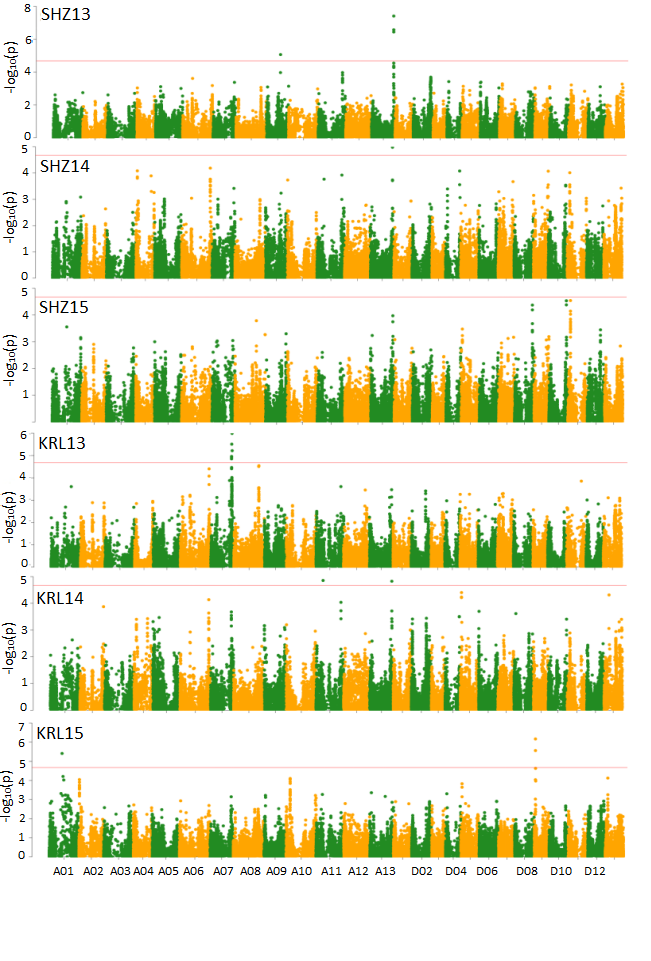

Supplement: Figure S6 — Manhattan plots showing the GWAS for FU in six environments. The horizontal line indicates the threshold (–log10P > 4.68). [file Image_6.TIF]

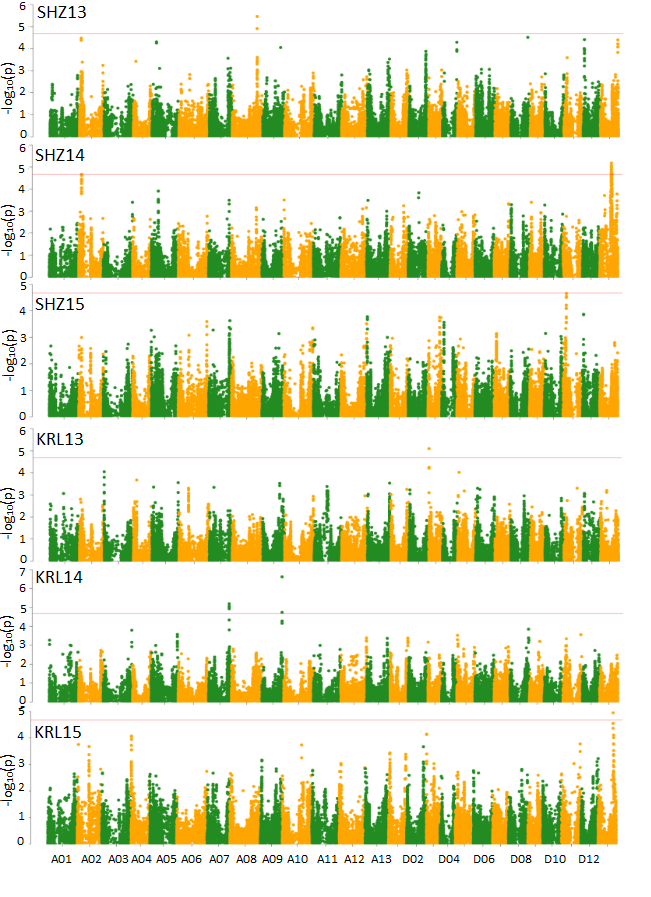

Supplement: Figure S7 — Manhattan plots showing the GWAS for FE in six environments. The horizontal line indicates the threshold (–log10P > 4.68). [file Image_7.TIF]

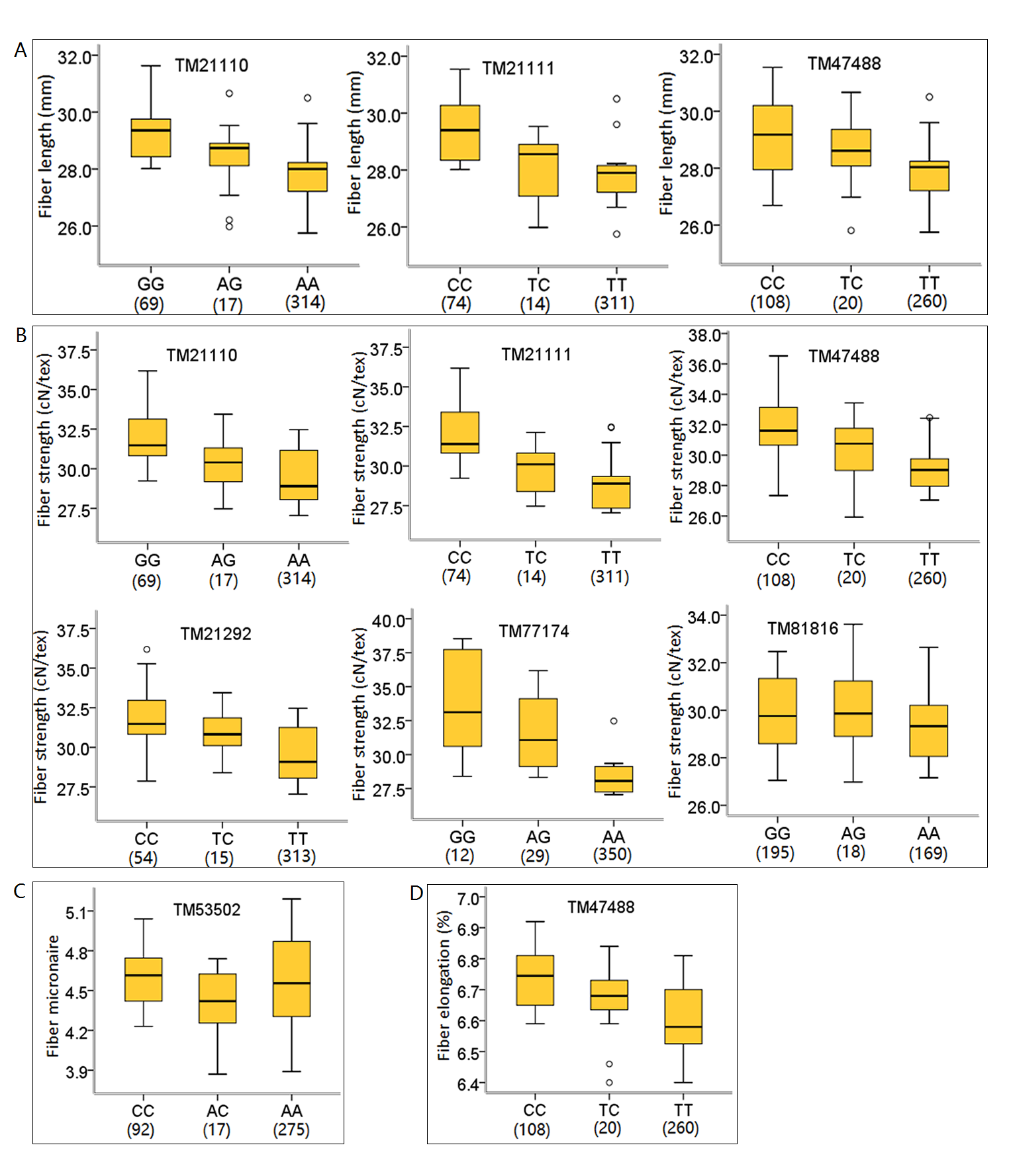

Supplement: Figure S8 — Boxplots depicting the genetic effects of SNPs with significant associations with fiber quality traits. (A) Boxplot diagram depicting the genetic effect of three significant SNPs to FL. (B) Boxplot diagram depicting the genetic effect of six significant SNPs to FS. (C) Boxplot diagram depicting the genetic effect of one significant SNP to FM. (D) Boxplot diagram depicting the genetic effect of one significant SNP to FE. The box shows the lower quartile, median and upper quartile values, and the whiskers show the range of phenotypic variation in the population. [file Image_8.TIF]

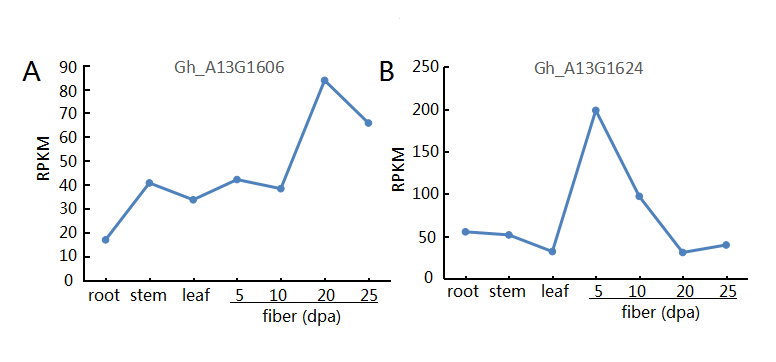

Supplement: Figure S9 — Expression levels of two genes associated with fiber quality traits. (A) Gh_A13G1606, (B) Gh_A13G1624. [file Image_9.TIF]
